# The frequency and magnitude of growth failure in a group of HIV-infected children in Cameroon

**Published:** 2012-01-25

**Authors:** Andreas Chiabi, Jacqueline Lebela, Marie kobela, Lawrence Mbuagbaw, Marie Thérèse Obama, Tetanye Ekoe

**Affiliations:** 1 Yaoundé Gynaeco-Obstetric and Pediatric Hospital/Faculty of Medicine and Biomedical Sciences, University of Yaoundé I, Cameroon; 2 Faculty of Medicine and Biomedical Sciences, University of Yaoundé I, Cameroon; 3 Mother Child Centre of the Chantal Biya Foundation/Faculty of Medicine and Biomedical Sciences, University of Yaoundé I, Cameroon; 4 Bafut District Hospital, North West Region, Cameroon; 5 University Teaching Hospital/ Faculty of Medicine and Biomedical Sciences, University of Yaoundé I, Cameroon; 6 Faculty of Medicine and Biomedical Sciences, University of Yaoundé I, Cameroon

**Keywords:** Anthropometry, HIV infected children, growth, Cameroon

## Abstract

**Background:**

Growth impairment is a major manifestation of HIV infection in children and has been implicated as a major contributor to both morbidity and mortality. This study the first to be done in this setting, was aimed at comparing the growth of HIV infected children to that of non-infected children in two referral health facilities in Yaoundé, Cameroon.

**Methods:**

A prospective case control study was carried out on 39 HIV infected children in two referral hospitals and followed up for a period of 12 months. Anthropometric measurements were taken and the sociodemographic variables of mothers and infants noted. Thirty nine infected children (mean age 45.3 months ± 41.6 SD) were age and sex matched with 39 non-infected children (mean age 44.4 ± 40.7 months).

**Results:**

Out of the 39 infected children, 26 (66.7%) had at least one of the three anthropometric indices (weight for height, weight for age, height for age) Z scores less than −2. Throughout follow-up, 20.5% of the infected children were wasted (weight to height Z score < −2) versus none in the control group, 56.4% underweight (weight for age Z score < −2) in the infected versus 2.6% in the control group, and 51.3% stunted (height for age Z score < −2) in contrast to 5.1% in the control group.

**Conclusion:**

This study demonstrated that wasting; underweight and stunting are common findings in HIV- infected children, thus stressing the importance of anthropometry in the routine care of these children.

## Background

Growth retardation is common with HIV infection and is an independent risk indicator for mortality, occurring in approximately 50% of children surviving to the age of 5 years [1]. In HIV infected toddlers and children; growth, psychomotor development, infectious complications and death are the four main measures used to define the clinical evolution and outcome of the disease [2]. Several studies done around the world using anthropometric indices (weight for age, height for age and weight for height) have demonstrated growth failure in HIV infected children [1–10].

In Cameroon no studies have been done to assess growth of HIV infected children. This study was undertaken to fill this gap by determining the frequency and magnitude of growth failure in HIV infected children in Cameroon.

## Methods

This was a prospective case control study carried out over 14 months from September 2008 to October 2009 in two referral hospitals in Yaoundé, the Mother and Child Center of the Chantal Biya Foundation and the Yaoundé University Teaching hospital. Children aged 6 weeks to 15 years with confirmed HIV infection were included. Controls were recruited during routine vaccination sessions in the two hospitals and were matched for age and sex with the HIV-infected children. The control group was similar in all respects to the exposed cohort with the exception that they were HIV negative (Table 1). The sample size was calculated from the Fleiss and Fless formula estimating the prevalence of malnutrition in HIV infected children in Cameroon at 30% [11]. This calculation gave us a sample of 36 children per group and a total of 72 children.

**Table 1:** The frequency and magnitude of growth failure in a group of HIV-infected children in Cameroon - Study population

Characteristics	Infected	Non infected
Number of cases	39	39
Males	20	20
Females	19	19
Number of measurements	162	143
Age < 18 months	14	14
Age > 18 months	25	25

The children in group 1 were either born from HIV infected mothers or clinically suspected to be infected and subsequently confirmed by a positive polymerase chain reaction (PCR) test in those less than 18 months, or enzyme linked immunosorbent assay (ELISA) confirmed by western blot (in those greater than 18 months). None of the children in group 1 was on antiretroviral therapy when recruited into the study but were later placed on antiretroviral drugs because they were eligible for treatment. Those in group 2 were non-infected (negative PCR or ELISA as in group I), healthy and without chronic disease. The ELISA serologies were done at the “Centre Pasteur” of Yaounde and the PCRs at the Chantal Biya International Research Centre on HIV after informed consent from the parents and pre-test counseling. Excluded from the study were the following categories of children: a) children having a chronic disease (e.g. sickle cell anemia, malignancies) which could have an independent effect on growth, b) children less than 6 weeks of age (because PCR testing is not routinely done in children born from HIV positive mothers before that age in Cameroon); c) children older than 15 years; children whose parents chose not to participate in the study.

Parents were briefed on how the study was to be carried out and on the necessity to respect future appointments and follow up of the children. The parents also benefited from nutritional counseling according to the infant nutrition recommendations of the Cameroon Ministry of Public Health [12].

Weights were determined using a Seca-Sauglingwage baby scale for toddlers and the Health Scale-Mic balance for older children to the nearest 0.1kg. Heights were measured standing for children older than 24 months or supine for younger children, with an appropriate Seca height gauge. The mid upper arm circumference (MUAC) was measured at the mid upper arm point of the child's left upper arm to the nearest 0.1 cm with a non-stretch tape with the elbow flexed at 90°. The mid upper arm point is half the distance between the tip of the shoulder blade and tip of the elbow). The children were undressed during these measurements.

Repeat evaluations were at 3, 6, 9 and 12 months. Data were analyzed with the Epi info 3.5.1 software and the CDC (Center for Disease Control) software 2000. The weight-for-height, height-for-age, weight-for-height Z scores, MUAC, and head circumferences were compared between the two groups. The Chi square and Student's t-tests were used to determine the degree of significance. P values less than 0.05 were considered statistically significant.

Authorization for this study was obtained from the authorities of both hospitals and ethical clearance from the ethical committee of the Cameroon Ministry of Public Health.

## Results

The study matched 39 infected cases for age and sex to 39 non infected controls. An effort was made to exclude confounding factors such as the presence of chronic disease in the control group. All anthropometric indices apart from the age and the BMI were significantly more affected in infected children. The weight for age index (WAZ) was the most affected. Their characteristics are summarized in Table 1 and Table 2.

**Table 2:** Age and anthropometric characteristics of the study population. Data are presented as means + standard deviations

Parameters	Infected n=39	Non Infected n=39	Student's test
Age (months)	45.3 ± 41.6	44.4 ± 40.7	0.81
Weight (kg)	13.57 ± 8.82	17.03 ± 8.04	< 0.001
Height (cm)	94.04 ± 61.22	98.1 ± 24.9	< 0.001
Weight/Age (SD)	−1.9 ± 2.1	0.53 ± 1.12	< 0.001
Weight/Height (SD)	−1.2 ± 2.5	0.77 ± 1.1	< 0.001
Height/Age (SD)	−1.5 ± 1.4	0.36 ± 1.2	< 0.001
BMI	15.7 ± 2.09	15.7 ± 1.2	0.63
MUAC (cm)	14.4 ±2.8	17.1 ± 1.8	< 0.001
HC (cm)	47.57 ± 4.6	48.9 ± 5.2	< 0.001

MUAC= mid upper arm circumference; BMI= body mass index; HC= head circumference; M= mean; SD= Standard deviation

### Z scores of anthropometric indices at recruitment

In the infected children, 20.5% had a WHZ< −2 versus 0.0% in the non-infected group with a significant difference. 51.3% of infected children had a HAZ index < −2 versus 5.1% in the non-infected children. 56.4% of infected children had a WAZ < −2 score compared to 2.6% in non-infected children. The difference in the z scores of the different indices was significant (Table 3).

**Table 3:** Distribution of the Z scores of anthropometric indices

Anthropometric Indices	Infected (n=39) %(Number)	Non infected(n=39) % (Number)	P
**WHZ**			
< -2	20.5(8)	0.0 (0)	(0.002)
> -2 SD	79.5 (31)	100 (39)	(0.002)
**HAZ**			
< -2 SD	51.3 (20)	5.1 (2)	(0.000)
> -2 SD	48.7 (19)	94.9 (37)	(0.000)
**WAZ**			
< -2 SD	56.4 (22)	2.6 (1)	(0.000)
> -2 SD	43.6 (17)	97.4 (38)	(0.000)

WHZ=weight for height Z score, HAZ= height for age Z score, WAZ = weight for age Z score, SD= standard deviation

### Mean Z scores by age group over the 12 months follow- up period

The WAZ was lowest in the 27 months age group with a mean of -5.9 in the infected children with a significant difference in both the infected and non-infected groups. The WHZ was also lowest in the 27 months age group with a mean of -5.4 in the infected children and a significant difference in both groups. For the HAZ, the most affected were the 48 and 127 months age groups with a mean of -2.7 for the infected group (Table 4).

**Table 4:** Mean Z score by age group over the 12 months follow up period

Age group (months)	WAZ score		HAZ score		WHZ score	
	Infected	Non infected	Infected	Non infected	Infected	non Infected
	
	N(M)	N(M)	N(M)	N(M)	N(M)	N(M)
3	14(−2.4)	12(0.3)*	14(−2.4)	12(−0.7)	14(−0.8)	12(1.4)*
4	5(−2.6)	4(3.8)	5(−1.1)	4(1.1)*	5(−2.3)	4(3.3)*
5	16(−2.6)	13(1.0)*	16(−0.9)	13(−0-04)	16(−3.0)	13(0.5)*
6	5(−1.7)	3(1)*	5(−2.6)	3(−1)*	5(0.6)	3(2.5)*
9	7(−4.0)	5(0.9)*	7(−2.0)	5(0.9)*	7(−2.9)	5(0.8)*
14	4(−1.5)	4(0.06)*	3(−1.0)	4(−0.4)*	3(−1)	4(0.9)*
16	10(−3.2)	8(1.4)*	10(−2.3)	8(1.1)*	10(−1.3)	8(1.5)*
17	3(−0.7)	3(−0.2)	3(−1.1)	3(0.2)*	3(0.3)	3(−0.7)*
18	9(−3.5)	9(0.4)*	9(−2.4)	9(0.2)*	6(−1.7)	9(0.6)*
23	5(−1.9)	5(0.6)*	5(−2.5)	5(0.4)*	5(−0.8)	5(0.4)
24	3(0.8)	3(1.1)*	3(−0.4)	3(0.4)*	3(1.5)	3(1.2)
25	5(−0.7)	5(−0.2)	5(−1.4)	5(0.4)*	5(0.1)	5(−0.7)*
27	2(−5.9)	1(1.5)*	2(−2.1)	1(0.1)*	2(−5.4)	1(0.2)*
32	3(−0.5)	3(0.6)*	3(−0.9)	3(1.5)*	3(0.0)	3(−0.3)
34	9(−0.7)	9(0.4)	9(−0.6)	9(0.7)	9(−0.4)	9(0.0)
35	4(−1.4)	4(−0.9)	4 (−1.3)	4(0.4)	4(−1.0)	4(−1.7)
36	4(−0.8)	3(1.6)*	4(−2.4)	3(1.7)*	4(0.9)	3(1)
45	4(−0.1)	4(0.7)*	4(−0.3)	4(1.8)*	4(−0.0)	4(−0.5)
47	5(−1.8)	5(−0.1)*	5(−1.9)	5(−0.5)*	5(−0.8)	5(0.2)
48	5(−1.4)	4(0.3)*	5(−2.7)	4(0.8)*	5(0.4)	4(−0.1)*
67	4(−2.8)	5(1)*	4(−1.3)	5(2.1)*		
68	4(−1)	4(0.5)*	4(−0.9)	4(−0.3)	4(−05)	4(1.1)*
88	4(−1.1)	4(0.6)*	4(−1.5)	4(0.8)*		
108	4(−0.6)	4(−0.0)*	4(0.6)	4(0.7)		
122	5(−0.3)	4(0.2)*	5(−0.3)	4(−0.0)		
127	5(−2.8)	4(−0.6)*	5(−2.7)	4(0.3)*		
130	3(−1.4)	3(−1.2)	3(−1.7)	3(−1.3)*		
138	6(0.1)	5(−0.5)*	6(−0.1)	5(0.2)		

N=number of measurements, M= mean Z scores

* significant difference

### Evolution of the anthropometric indices over the follow-up period

**Figure 1:**
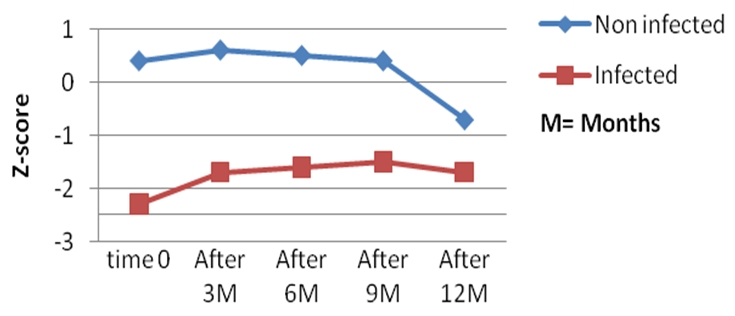
Evolution of the weight for age index (WA) Z scores during the 12 months follow up

**Figure 2:**
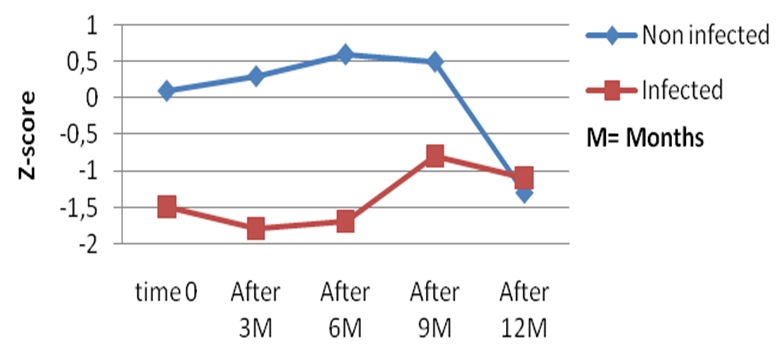
Evolution of the height for age (HA0 Z scores during the 12 months follow up

**Figure 3:**
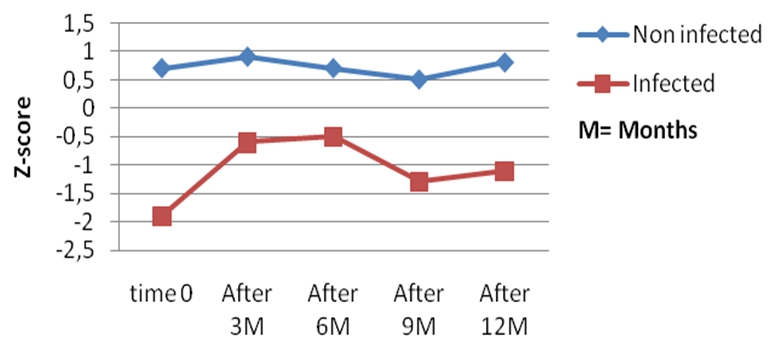
Evolution of the weight for height (WH) Z scores during the 12 months follow up

All the infected children had low WAZ, HAZ, and WHZ scores compared to the non-infected controls all through the 12 months follow up period (Figure 1, Figure 2, and Figure 3). Twenty-six had at least one anthropometric index (WHZ, WAZ, HAZ) < −2 Z score, giving an incidence of 66.7% of malnutrition, and 12 (30.7%) of the infected children had all 3 anthropometric indices less than −2 Z scores, whereas none of the children in the control group had an anthropometric index less than −2 Z-score at presentation and all through the follow-up period.

## Discussion

The mean age was insignificantly lower in the control group than in the infected children. Beau et al [13] in Cote d'Ivoire found a higher mean age of 26.1 months ± 12.6 in infected children than in controls (21.9 months ± 4.6) but the difference was not significant in that study, as well. All 39 infected children had vertical transmission of HIV infection, and 5 had failure of preventive treatment for mother-to-child transmission. Except for a 7 month old child who was lost to follow-up, all the infected children were placed on antiretroviral therapy because they were eligible for treatment. 25(64%) patients were infected with HIV 1 and 14(36%) were infected with HIV 2.

Twenty percent of the infected children had a mean weight for height Z-score less than −2 versus 0% in the controls. The mean Z score was −1.2 ± 2.5 in the infected group versus 0.77 ± 1.1 for the controls with a statistically significant difference. Beau et al. reported a mean WHZ of −3.9 ± 0.8 in the infected children against −3.8 ± 0.9 for the non-infected [13]. This is explained by the fact that in our study the infected children were recruited at different clinical stages whereas Beau et al studied a population of malnourished children. The 27 months age group was the most affected with a mean Z score of 5.4, followed by the 5 and 9 months age groups with −3 and −2.9 Z scores respectively. Apart from these 3 categories, the results were similar to those of non-infected children. Lepage et al, also had similar results in a study of HIV infected school children in Kigali, Rwanda [3], whereas, Bailey et al in Kinshasa [5] had no children with a WHZ score of less than -2. In a study to assess the growth parameters of infants born to HIV- infected mothers, Venkatesh et al noted that 29% of them had weight for length Z scores < -2 [9].

Our study found 3 severely affected age groups (5, 9, and 27 months) probably because these children were recruited at advanced HIV disease stage.

The weight to height index has long been considered an important indicator in evaluating health status in children [14, 15]. The pathophysiology of wasting in HIV infected children remains unclear. According to Yip et al, wasting is usually associated with a severe acute recent illness and generally reflects acute malnutrition and caused mainly by persistent diarrhea[16]. Wasting in HIV infected persons could be due to many factors associating low oral intake, malabsorption, endocrine disorders and other metabolic disorders, such as the effect of tumour necrosis factor (TNFa) [17].

A low weight for age was observed in the infected group all through the study period compared to the controls, with the Z score below 0. The 27 months age group was the most affected. Fifty six percent of the infected children were underweight with a WAZ score less than −2 against 2.6% in the controls. The values of the mean WAZ scores were lower than those observed by Beau et al in Cote d'Ivoire [13]. This is explained by the fact that we had 14 children less than 18 months old with two positive HIV results and, according to recent recommendations of the Cameroon Ministry of Public Health, are considered infected whatever the clinical or immunological stage. Amongst these 14 children, the mothers of 5 had received antiretrovirals during pregnancy in an attempt to prevent mother-to-child HIV transmission.

Bailey et al, observed that infected children from 14, 16, and 18 months age groups were underweight with a Z score less than -2 [5]. A similar finding was made by Arpadi et al [1]. In Rwanda, significant low weight for age Z scores were noted between 9 and 12 months of age [3], in South Africa by 3 months of age [9], and in Zambia the WAZ scores declined precipitously between 4.5 and 15 months [18]. According to Berhane et al, seropositive children with a weight for age Z score less than −1.5 in the first year of life had 5 times more the risk of dying before the age of 25 months than seronegative controls [19]. In 56.4% of our children the WAZ scores were less than -2 because most of our children were recruited at immunological stage 4 and clinical stage III of the WHO classification [20].

In 51.3% of the infected children, the height for age Z score (HAZ) was less than -2 indicating stunting. This figure is somewhat less than the 66% observed by Beau et al in their study in Cote d'Ivoire [13]. This finding confirms the fact that stunting is frequent in malnourished HIV infected children. However, in the study of Beau et al, the rate of stunting was similar in both the infected and non-infected groups indicating in that study that HIV infection was not a discriminating factor for HIV infection. The mean HAZ score in our study (−1.5) is comparable to that observed by Shet et al (−1.7) in India [21], and less impaired than those reported by Beau et al (−2.5) [13], and Arpadi et al (−2.2) [1].

The most affected age groups reported were those of 48 and 127 months. Lepage et al observed HAZ scores less than -2 at 9 to 48 months of age [3]. They also found a constantly low HAZ scores all through the study period in the infected children compared to controls. A low height for age (stunting) might be a consequence of insufficient feeding, an increase in the frequency of infections, or both.

The mean BMI of the infected children in our study was 15.7 ± 2.09 compared to 10.7 ± 1.3 in the study of Beau et al[ (13], without a significant difference between the infected and control groups in both studies. The BMI increases rapidly during childhood with a peak at about 12 months of age, then decreases to a plateau and remains stable between 5 to 6 years in both boys and girls [22]. The gradual decrease of this index has been correlated with different stages of the disease in HIV infected children [2, 13]. It has also been found to be an indicator of poor prognosis in malnourished seropositive children [13, 23].

### Limitations of the study

This study was limited by the fact that confounders as maternal socio-economic status, gestational ages, use of anti-retroviral therapy, concomitant infections and feeding practices were not studied. Also the sample size was not significantly powered to detect significant differences in the two groups. All these would have contributed to the validity of the reported findings.

## Conclusion

Growth retardation is commonly associated with HIV infection in children but little is documented about it in Cameroonian children. Our study demonstrates that HIV infected children in our study population have growth failure with stunting (51.3%), underweight (56.4%), and wasting (20.5%) despite antiretroviral treatment. It demonstrates significantly low WAZ, WHZ, HAZ indices all through the 12 months follow-up. Such a poor trend of anthropometric indices likely increases the already significant risk of mortality in these children. This study thus, indicates the importance of taking anthropometric measurements by health care workers in the routine care of HIV infected children so that abnormalities could be detected early enough and appropriate management sought to avert significant morbidity and mortality.
